# Inferring the presence of aflatoxin-producing *Aspergillus flavus* strains using RNA sequencing and electronic probes as a transcriptomic screening tool

**DOI:** 10.1371/journal.pone.0198575

**Published:** 2018-10-16

**Authors:** Andres S. Espindola, William Schneider, Kitty F. Cardwell, Yisel Carrillo, Peter R. Hoyt, Stephen M. Marek, Hassan A. Melouk, Carla D. Garzon

**Affiliations:** 1 Department of Entomology and Plant Pathology, Oklahoma State University, Stillwater, Oklahoma, United States of America; 2 National Institute for Microbial Forensics and Food and Agricultural Biosecurity (NIMFFAB), Oklahoma State University, Stillwater, Oklahoma, United States of America; 3 Department of Biochemistry and Molecular Biology, Oklahoma State University, Stillwater, Oklahoma, United States of America; University of Nebraska-Lincoln, UNITED STATES

## Abstract

E-probe Diagnostic for Nucleic acid Analysis (EDNA) is a bioinformatic tool originally developed to detect plant pathogens in metagenomic databases. However, enhancements made to EDNA increased its capacity to conduct hypothesis directed detection of specific gene targets present in transcriptomic databases. To target specific pathogenicity factors used by the pathogen to infect its host or other targets of interest, e-probes need to be developed for transcripts related to that function. In this study, EDNA transcriptomics (EDNAtran) was developed to detect the expression of genes related to aflatoxin production at the transcriptomic level. E-probes were designed from genes up-regulated during *A*. *flavus* aflatoxin production. EDNAtran detected gene transcripts related to aflatoxin production in a transcriptomic database from corn, where aflatoxin was produced. The results were significantly different from e-probes being used in the transcriptomic database where aflatoxin was not produced (atoxigenic AF36 strain and toxigenic AF70 in Potato Dextrose Broth).

## Introduction

Maize [1], peanuts [2], tree nuts, dried spices [3] and cottonseed [4] are crops that can be infected during the pre-harvest, post-harvest and/or storage period with *Aspergillus flavus* Link. This fungus produces polyketide secondary metabolites named aflatoxins. Among the four known aflatoxins (B_1_, B_2_, G_1_, G_2_), B_1_ has been of special interest to food biosecurity due to its toxicity and potent carcinogenic properties [5]. *A*. *flavus* is a ubiquitous saprophytic ascomycete fungus grouped in the *Aspergillus* section Flavi, species with aflatoxin-producing strains including *A*. *flavus*, *A*. *parasiticus* and *A*. *nomius* [6,7].

Aflatoxin is produced through the interaction of approximately 25 genes in a cluster cascade [8–10]. Regulatory genes for the cluster are *aflR* and *aflS* (*aflJ*), where *aflR* encodes for a transcriptional factor of the type Zn(II)_2_Cys_6_ which binds promoter regions of many aflatoxin genes [11–14]. In contrast, aflS (*aflJ*) regulates aflatoxin production through binding and activating aflR [15]. Some strains of *A*. *flavus* do not produce aflatoxin and these have been shown to have deletion mutations and/or single nucleotide polymorphisms (SNPs), that have been identified by amplifying 32 specific regions of the cluster [16]. Each PCR amplification was designed to flank biosynthetic gene regions in the aflatoxin gene cluster. Determining the presence/absence of the amplicon allowed confirmation of the absence of genes crucial for aflatoxin production in atoxigenic strains [16]. On the other hand, atoxigenic strains having the complete aflatoxin gene cluster can have SNPs which have either decreased or completely eliminated aflatoxin production [17]. Callicott and Cotty [18] have begun to use cluster amplification patterns (CAPS) to evaluate *A*. *flavus* populations based on genotype.

Aflatoxin contamination in food is highly regulated in multiple countries, consequently increasing management costs and final product price [19–21]. In the United States alone, the maximum allowed concentration of aflatoxin in food for human consumption is 20 ppb, as dictated by the U.S. Food and Drug Administration (FDA). Appropriate and accurate aflatoxin testing is necessary to avoid human and animal exposure to aflatoxins in food and feed respectively. Among the most used techniques for aflatoxin detection and quantification are thin layer chromatography (TLC), high-performance liquid chromatography (HPLC), enzyme-linked immunosorbent assay (ELISA) and fluorometry [19], however, there are limitations in all of these for rapid testing. Industry costs for testing crops for aflatoxins in the United States alone have ranged from $30 to $50 million per year at approximately $10 to $20 per sample tested [21].

We propose that cost/sample test can be reduced when the viability (active mRNA transcripts) and presence of toxigenic strains (unique up-regulated genes belonging to toxigenic strains) can be accurately inferred using sequencing technology. Our research indicates that testing for aflatoxin-associated upregulated gene sequences in grain populations can provide a fast and potentially low-cost screening tool for determining presence and activity of toxigenic *A*. *flavus* strains. It is becoming very inexpensive to sequence [22]. Therefore, sequencing the whole transcriptome (metatranscriptome) of the plant matrix or soil niche without the need for isolation, culturing, genome assembly or toxin testing will be significantly faster than current methods. The metatranscriptome can then be screened *in silico* to detect the presence of toxigenic gene sequence up-regulation. Although we have not shown data about how *A*. *flavus* population ratio fluctuations can be measured by this technique, the current research sets a baseline and an introduction to the use of EDNAtran as a mean of inferring aflatoxin. However, we believe that we might be able (in the near future) to use mRNA transcripts to both infer aflatoxin production and assess shifts in *A*. *flavus* population toxigenic potential.

Previously, various approaches have been developed to use metagenomes in ecology studies and determine the microbial profile of natural ecosystems [23,24]. Tools to detect microbes at the species/isolate level in agricultural ecosystems are known [25–27]. However, none of them has addressed the detection of gene activity and upregulation in agroecosystems. For example, the ecological and evolutionary drivers of *A*. *flavus* population toxigenicity in the soil are only surmised. A quick and easy research tool to evaluate when and under what conditions up-regulation of toxin production begins could help understand population drivers in the soil and plant environments. E-probe Diagnostic for Nucleic acid Analysis (EDNA) was designed to detect viruses, bacteria, fungi and oomycete plant pathogens by using species-specific markers named e-probes [25,26,28]. E-probes are carefully designed unique DNA signatures of plant pathogen genomes, validated for sensitivity, specificity, and limit of detection. E-probes are used *in silico* to detect presence or absence of one or multiple pathogens in raw sequencing data [25,26]. Here we modified EDNA to be used as a gene functional analysis and detection tool to infer the presence of aflatoxin and potentially toxigenic *A*. *flavus* strains. EDNA transcriptomics—a modification of the original EDNA’s bioinformatic pipeline [25]—was designed to incorporate functional genome annotations on the e-probe design as well as on the detection pipelines. EDNAtran is a theoretical approach that is being tested for the first time with *A*. *flavus* and could be extended to detect metabolic functions associated with pathogenicity in other host-pathogen systems. Detecting metabolic functions that could potentially lead to plant disease is crucial to incorporate proper and timely management practices in agroecosystems.

## Materials and methods

### Fungal isolates and culture methods

*A*. *flavus* strains were obtained as freeze-dried (AF36; ATCC 96045; atoxigenic) and frozen (AF70; ATCC MYA-384; toxigenic) cultures from ATCC (Manassas, VA). AF36 was reactivated by rehydration, adding 500 μL of sterilized distilled water inside the vial. Subsequently, 100 μL of the re-suspended AF36 was plated on Malt extract agar Blakeslee’s formula (MEAbl) and incubated at 31°C in darkness until mycelium was developed (72 hours), according to ATCC instructions. AF70 was thawed for 5 minutes, directly plated onto Malt extract agar, and incubated at 25°C in darkness until mycelium was developed (72 hours), according to ATCC instructions. Agar plugs with actively growing mycelia were re-plated in MEAbl agar and incubated at their optimal temperatures in the dark until extensive conidial development (5 days) was observed. The cultures (AF36 and AF70) containing extensive conidia growth were used to inoculate ground corn and Potato Dextrose Broth (PDB).

Corn substrate was prepared using dried corn kernels (*Zea mays*). Kernels were weight (20g) and ground (using a coffee grinder Mr. Coffee Precision Coffee Grinder IDS77) until obtaining pieces with the approximate texture of coarse sand (0.5-1mm in diameter). The coarse grains were autoclaved (dry cycle) for 20 minutes in polycarbonate containers (Magenta GA-7, Plantmedia, US) and its humidity was adjusted to keep between 25–33% w/v (Modified from Woloshuk, Cavaletto, and Cleveland 1997 [29]).

Ground corn kernels and PDB media were inoculated with conidial suspensions obtained by washing *A*. *flavus* MEAbl plates with 2 mL of sterile distilled water. Conidia collected (2 mL) were then added to a single vial containing 4mL of distilled water for a final dilution of 3:1 v/v (Spore suspension was not quantified). Six mL of spore suspension was used to inoculate each replicate (20 g of ground corn and PDB). The ground grain was inoculated with the *A*. *flavus* suspension in polycarbonate containers and homogeneously mixed by rolling the containers to allow uniform distribution of the conidia. Similarly, 250 mL flasks containing 44mL of PDB were inoculated with 6 mL of *A*. *flavus* spore suspension. The containers and PDB plates were incubated at 31°C in the dark for 10 days. The isolates of *A*. *flavus* AF70 and AF36 exhibit different growth patterns and morphology on the different media (PDB and ground corn). Primarily sclerotia production was observed in AF70, whereas AF36 produced conidia in all media.

### Aflatoxin extraction and quantification

Aflatoxin was extracted using 70% methanol. Briefly, the ground corn (20 g) was suspended in 100 mL of 70% methanol and mixed vigorously for 2 minutes. Then, the extract was filtrated through a Whatman # 1 filter paper and methanol was collected for testing. Aflatoxin B1 was quantified using the Rapid Aflatoxin B1 ELISA kit (Sigma-Aldrich) following the manufacturer’s protocol. The ELISA plate was read using an ALx800 plate reader at 450 nm and a dose-response curve was created to calculate aflatoxin production. The Limit of Detection (LOD) was determined following the manufacturer’s guidelines and we used autoclaved, non-inoculated ground corn as a negative control for corn and non-inoculated PDB as negative control for PDB. We calculated the LOD for ground corn and PDB by using the mean concentration plus two standard deviations of three measurements.

### RNA extraction and sequencing

Ground corn kernels inoculated with the AF70 and AF36 strains produced extensive conidia, which were suspended by gently adding 10 mL of sterilized water (plus Tween 20) to the magenta containers. The containers were shaken gently to homogenize the spores and then 1 mL of the spore suspension was obtained and added to a capped 2mL tube containing silica beads. The conidia cell walls were disrupted by shaking the 2mL tubes using a bead beater (2 cycles of 20 seconds). The lysate was then transferred (500 μL) to a column of the Qiagen RNeasy Plant Mini Kit for RNA extraction. On the other hand, for AF36 and AF70 growing on PDB, mycelia/spores were recovered by filtrating them using Whatman paper. 100 mg of mycelium/spores were weight and added to a column of the Qiagen RNeasy Plant Mini Kit to continue with the RNA extraction procedure. The RNA quality and integrity were assessed using a 2100 Agilent Bioanalyzer (Agilent Technologies) for 12 RNA extraction samples from AF36 and AF70. A sample (per strain) having RIN numbers higher than eight were selected for RNA sequencing. After quality control, RNA was sequenced using the Illumina HiSeq 2500 sequencer at the Core Facility of the University of Illinois at Urbana-Champaign, IL. The mRNA sequencing library was created with PolyA capture method per manufacturer’s protocol and the library was sequenced as single-end.

### Gene expression analysis

RNA sequencing reads from samples AF70-corn and AF70-PDB were mapped onto the *A*. *flavus* AF70 genome using STAR software [30] and bam binary files were obtained from sam files using SAMtools (http://samtools.sourceforge.net). Gene expression analysis was performed with DeSeq2 in R by comparing AF70 growing on two substrates (corn and PDB). The control was AF70 in PDB (non-conducive for aflatoxin production), and the treatment was AF70 in corn (conducive to aflatoxin production) [31]. Positive fold change (up-regulated) genes were selected using the log2 fold change metric obtained from the DeSeq2 analysis. Upregulated gene sequences having log2 fold changes greater than five were retrieved by an in-house Linux bash script and kept in a multi-fasta file for later e-probe design.

### E-probe design

The genome from *A*. *flavus* AF70 (JZDT00000000.1) [32] was obtained from Genbank. Sequences for the aflatoxin gene cluster of AF70 (AY510453) and AF36 (AY510455) were also retrieved from GenBank [33]. E-probes 80 nt long were generated using the e-probe pipeline for EDNAtran [25,26]. The aflatoxin gene cluster of AF70 was used as target sequence and the same gene cluster for AF36 was used as near neighbor sequence for developing highly specific e-probes. E-probe specificity was verified by local alignment of each e-probe with the intended target genome (AF70) using stringency of 100% identity and query coverage. Metadata information about the gene function and coordinates was also retrieved by the EDNAtran e-probe design pipeline. E-probes that belonged only to the up-regulated genes previously identified with DeSeq2 were selected. E-probe specificity with other toxigenic/atoxigenic strains was visualized with circos [34].

### Rapid assessment of active aflatoxin metabolic pathway using EDNAtran

EDNAtran was utilized with default parameters (percent identity and query coverage of 100%) to assess four transcriptomic databases (**Table 1**) which included toxigenic (AF70) and atoxigenic (AF36) *A*. *flavus* strains growing in conducive (ground corn) and non-conducive (PDB) environment for aflatoxin production. E-probes designed in up-regulated genes of the aflatoxin gene cluster of AF70 were utilized during this analysis. Hit frequencies of raw reads with e-probes were recorded for each of the four treatments. Data on hit frequencies were analyzed for variance by ANOVA to determine differences in the group. Significant hit frequency differences between treatments were determined with Tukey’s HSD test and pairwise T-test at P-value = 0.05.

**Table 1:** EDNA transcriptomics output table for the inference of aflatoxin in *A*. *flavus*.

Culture	Total reads	ARL	LRL	MRL	Probe length	TNP	HQM		
							Ev	NoEv	HSGM
AF70^a^	20657024	98.9	100	35	80	231	39	40	39
AF36^b^	22495368	99.0	100	35	80	231	2	2	2
AF36^a^	24134226	98.7	100	35	80	231	12	12	12
AF70^b^	24902500	98.9	100	35	80	231	231	231	231

ARL, average read length; LRL, largest read length; MRL, minimum read length; TNP, the total number of probes; HQM, high-quality matches; Ev, matches include e-value in the scoring method; NoEv, matches do not include e-value in the scoring method; HSGM, high scoring general matches.

^a^*A*. *flavus* strain growing on PDB.

^b^*A*. *flavus* strain growing on ground corn.

## Results

### RNA sequencing and gene expression analysis

**Fig 1:**
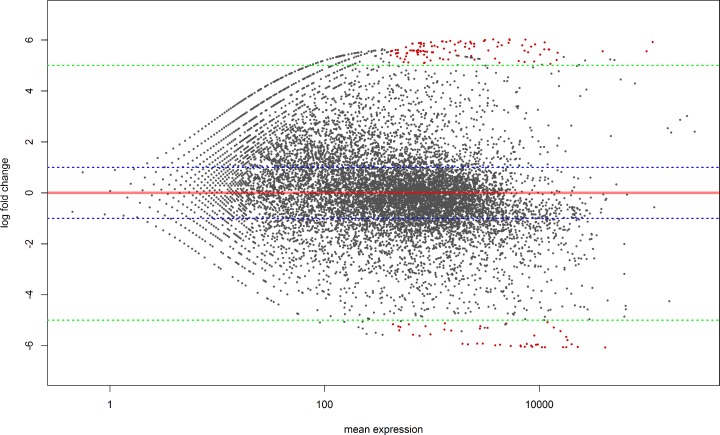
Mean average plot for RNA sequencing gene expression analysis. **Red line shows zero change in gene expression.** Blue dashed lines show no change in gene expression and green dashed lines show a five-fold change in gene expression. Red dots are genes that have been either up-regulated or down-regulated in *A*. *flavus* AF70 infecting ground corn. Gray dots depict genes that have not had enough statistical evidence to be assigned a gene expression fold change.

**Fig 2:**
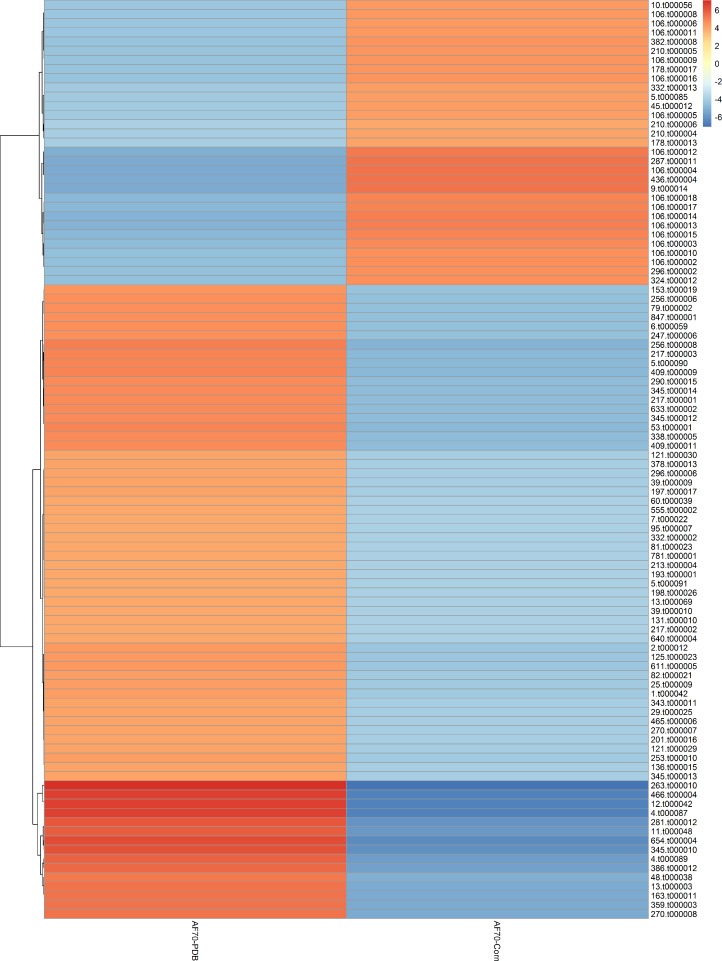
Hierarchical clustering map depicting *A*. *flavus* AF70 growing on PDA and ground corn. Gene expression fold change is differentiated by a color palette ranging from red (most up-regulated genes have plus six-fold changes) to blue (most down-regulated genes have minus six-fold change). Genes are clustered based on their gene expression fold change to facilitate gene co-expression analysis.

RNA extracted from AF36 and AF70 strains grown on PDB and ground corn yielded from 20 to 24,9 million reads per sequencing run (**Table 1**). The sequenced reads then were mapped to the *A*. *flavus* AF70 strain genome to retrieve information about potential up-regulation and down-regulation of genes by using STAR [30] and DESeq2. In total, 44 sequences were identified as up-regulated in AF70 on corn relative to AF36 and 129 were down-regulated (**S1 Table**). The majority of genes that were up-regulated but not associated with the aflatoxin gene cluster were responsible for housekeeping functions and a few played a role in melanin formation (data not shown). Of the upregulated sequences, only 17 out of 44 mapped to the aflatoxin gene cluster. From two to six gene fold changes were plotted in a hierarchical clustering heat map as well as in a MAPlot (**Figs 1 and 2**).

### E-probe generation for aflatoxin detection

**Fig 3:**
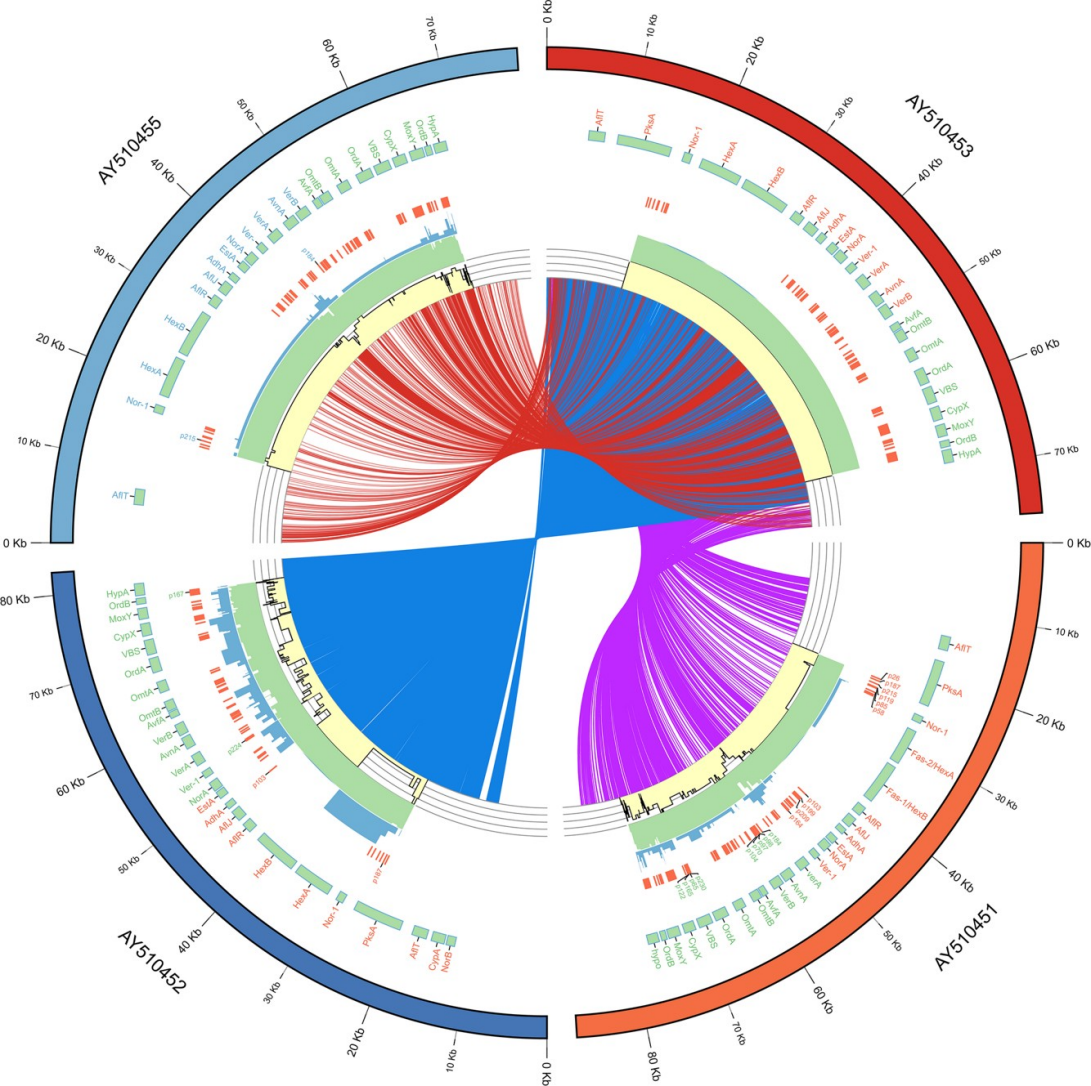
Circular representation of full-length aflatoxin gene clusters of AF70 (AY510453), AF36 (AY510455), AF13 (AY510451) and BN008 (AY510452). The colored outer ring represents each gene cluster for the four strains. The next ring (green) depicts mRNA coordinates and gene names associated to them. The following ring contains the e-probe localization on each strain cluster if when aligned separately. The e-probe ring contains all quality alignments. Additionally, it depicts the names of the e-probes that showed high-quality alignments (100% identity and query coverage). The following three rings are histograms representing mismatches, alignment lengths and percent identities of the e-probe alignments with each strain gene cluster. The inner lines depict Single nucleotide polymorphisms (SNPs) that were retrieved when comparing AF70 with the other three strains.

In total, 231 highly specific e-probes were generated on the up-regulated genes of AF70 that did not appear in AF36, indicating the production of aflatoxin specifically for AF70. The designed e-probes are available at our web portal http://www.edna2.okstate.edu when you sign up as a new user. Additionally, figures and data analysis can be reproduced using our GitHub repository (https://github.com/andrese52/EDNAtran). The e-probes could be utilized to detect most toxigenic strains that have similar SNPs patterns in the aflatoxin gene cluster (**Fig 3**). Further validation is required to include more toxigenic strains where they are sequenced as part of a metatranscriptome. Being this a proof of concept of EDNAtran, including more strains is out of the scope of this research. However, alignments of the e-probes with other toxigenic strains gene cluster have shown their usefulness for detecting other strains (**Fig 3**). However, separate validation must be performed for each strain. On the other hand, AF36 genome-wide e-probes were not generated because there is not a genome sequence available yet for that specific strain.

### Inferring aflatoxin production using EDNAtran in *A*. *flavus*

**Fig 4:**
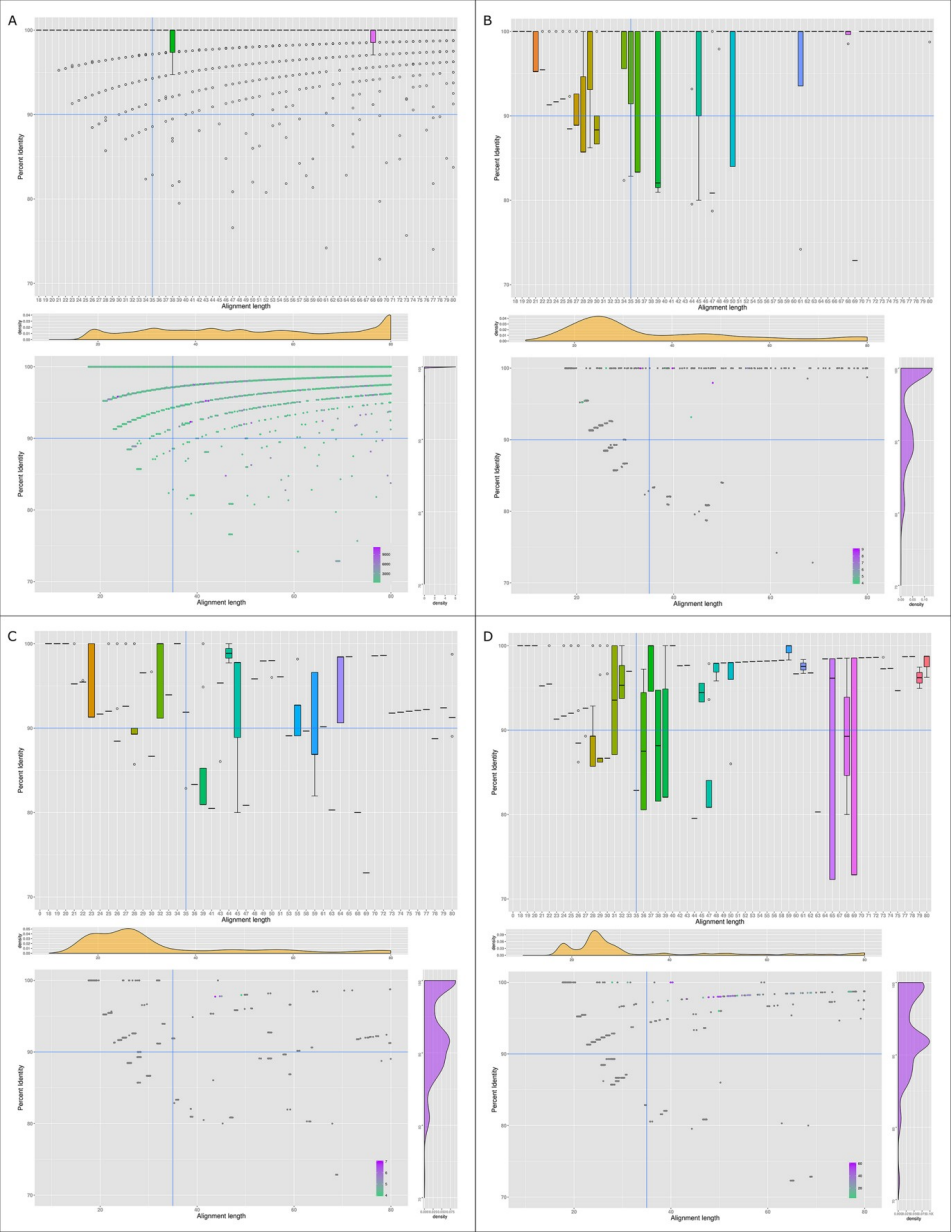
EDNA transcriptomic hits distribution and frequencies for *A*. *flavus* aflatoxin detection. (A and C) RNA sequencing of *A*. *flavus* AF70 and AF36 respectively growing on corn identified with 80-mer AF70 aflatoxin-specific e-probes. (B and D). RNA sequencing of *A*. *flavus* AF70 and AF36 respectively growing on PDB.

**Fig 5:**
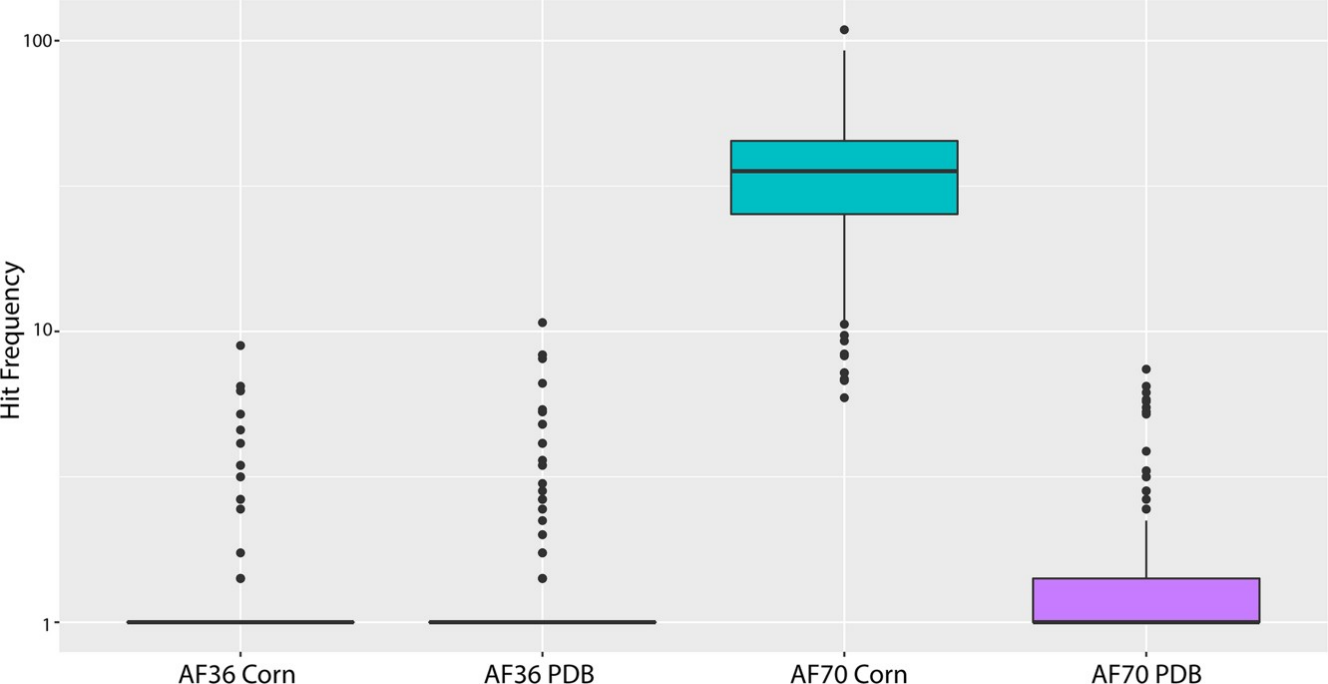
Hit frequencies of AF70 e-probes in RNA sequencing databases of *A*. *flavus*. Hit frequencies of the AF70 e-probes in the sequencing library of the atoxigenic strain (AF36) growing on both Corn and PDB. Similarly, hit frequencies of the AF70 e-probes in the sequencing library of AF70 growing on PDB and Corn. Differences in gene expression levels are directly correlated to hit frequencies. The dots outside the boxplots represent hit frequency outliers of single AF70 e-probes.

Aflatoxin is mainly produced in the presence of sucrose or oily substrates [35–37]. Aflatoxin B1 is produced in very high concentration when AF70 is growing in corn. Our measurements with a sandwich ELISA (Sigma-Aldrich) showed concentrations of aflatoxin B1 of 43.67 ppm when AF70 was growing in PDB and 455.79 when AF70 was growing in corn. Similarly, AF36 aflatoxin B1 production was measured and in all cases (Corn and PDB) the aflatoxin B1 concentration was 13.60 and 2.03 ppb respectively. The Limit of Detection (LOD) for the Rapid Aflatoxin B_1_ ELISA Kit calculated for both PDB and ground corn resulted in 1.72 and 13.05 ppb respectively. Similar aflatoxin B1 concentrations have been found for *in vitro* inoculation of corn with toxigenic strains [38,39]. As expected, 231 e-probes had hits creating High-Quality Matches (HQMs) in AF70-corn transcriptome datasets; meanwhile, AF70-PDB had only 39 HQMs. AF36-corn had only two HQMs and AF36-PDB had 12 HQMs (**Table 1**). EDNAtran discriminated between the transcriptomic databases with abundant aflatoxin production and the transcriptomes from low-toxin production based on EDNA eukaryotic metrics [26] (**Table 1**). However, to infer the presence of aflatoxin we have to use frequencies of hits as an indirect measure of aflatoxin production. It is expected that a high production of proteins (upregulated genes) involved in the synthesis of aflatoxin is associated with higher aflatoxin production. In this case, the number of times a read was mapped to an e-probe was recorded and counted without any limits (no normalization). A dot plot of alignment length vs. percent identity with marginal hit frequencies facilitates visualizing hit frequencies. Specifically for *A*. *flavus* AF70 in corn, it was observable that the hit frequencies were very high—around 9,000 hits per e-probe—when the alignments are above 90% identity and the alignment length was approaching to the total length of the e-probe (**Fig 4A**). Conversely, for AF70 in PDB and AF36, the marginal plots show a low frequency of hits when alignment lengths and percent identities were above the threshold of 35nt and 90% respectively (**Fig 4B–4D**). Frequencies of hit values were square root converted and statistically analyzed with ANOVA to compare all the samples/treatments (**Fig 5**).

**Fig 6:**
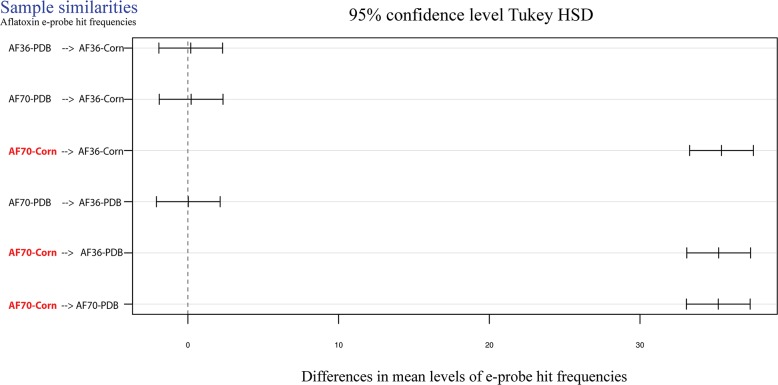
Post-hoc analysis of ANOVA using Tukey HSD with 95% of confidence for the inference of aflatoxin transcriptional activation using AF70 e-probes. Pairwise comparisons of hit frequency means between all sequencing libraries. Lines close to zero are sequencing libraries that had no difference in their hit frequency means while lines closer to 30 are sequencing libraries that had different hit frequency means. Red sequencing libraries (AF70-Corn) show the highest differences in mean levels which are above 30.

The ANOVA in the *A*. *flavus* experiment had a p-value lower than 0.05 which rejects the null hypotheses (all hit frequencies are equal); therefore, a post-hoc analysis was automatically performed using the Tukey HSD function in R. The post-hoc analysis and T-test for *A*. *flavus* showed that e-probes hitting on RNA sequencing databases obtained from *A*. *flavus* AF70 growing on ground corn were different from those of AF70 growing on PDB, and AF36 on corn and PDB (**Fig 6** and **Table 2**). In conclusion, EDNAtran was able to find statistically significant differences between the transcriptomic data set of the highly toxigenic sample, from the non-toxigenic samples, using 231 e-probes generated in this study.

**Table 2:** Pairwise T-test p-values comparing e-probe hit frequencies for *A*. *flavus* toxin detection analysis using the aflatoxin e-probes.

	AF36-Corn	AF36-PDB	AF70-Corn
AF36-PDB	1	NA	NA
AF70-Corn	3.72E-222^*^	8.38E-221^*^	NA
AF70-PDB	1	1	1.04E-220^*^

*Comparisons having p-values lower than 0.05 are considered significant.

## Discussion

EDNA has previously been proven to successfully detect a variety of plant pathogens from raw metagenomic databases [26,28,40]. DNA as the main source of identification has always been the gold standard for detecting organisms in a sample, although viability is not assessed. Therefore, the question about “dead or alive” is left undetermined unless the organism is isolated and cultured, or, transcriptome analysis is used as a complementary detection tool or, complementary molecular viability analysis is included [41]. Here we have been able to use e-probes to associate the production of a secondary metabolite with gene regulation. The amount of aflatoxin B_1_ measured under these experimental conditions when AF70 was inoculated in ground corn and PDB confirms that aflatoxin is produced and that EDNA transcriptomics can infer such production rapidly and without any assembly or read mapping to reference genomes. Using the same approach, AF36 showed trace amounts of aflatoxin in the ELISA test which are attributable to the LOD of the matrixes and possible cross contamination during the incubation period. Yet, EDNA transcriptomics also determined that aflatoxigenic genes were downregulated in AF36 in all experimental conditions.

Inspectors at international ports require a rapid detection method when decisions need to be done on site. EDNA has been considered a good candidate to be used as a diagnostic tool in ports of entry, due to its multiplexing capacity and rapidness. Yet, EDNA does not include an analysis of pathogen viability. If DNA-based detection (metagenomic analysis) is positive and viability needs to be addressed, the use of additional tests is not a viable approach for perishable or time-sensitive shipments. Using RNA sequencing and relative quantification of active genes is ideal to infer the viability of plant pathogens. The use of EDNA transcriptomics to infer the production of aflatoxin is a first attempt to introduce a novel strategy by using new sequencing technologies to identify actively metabolizing plant pathogens. The use of e-probes that are designed on up-regulated genes incorporates an advantage to EDNA transcriptomics over other tools that use RNA sequencing to assess gene expression [31]. The advantage of EDNA transcriptomics over other methods of transcript frequency inference and calculation is that the time-consuming map against the reference genome is not necessary. Instead, we align the sample reads to the highly specific e-probes, which are designed for known up-regulated genes. Directing the analysis to genes that are known to be up-regulated reduces the analysis time tremendously since a mapping against a whole genome is no longer necessary. Where needed total nucleic acids (DNA and RNA) can be extracted from the sample of interest to perform both pathogen detection and gene activity.

Although most of the potential controlled inputs must be maintained constant, the sample matrix could contain fungal biomass, spores or sclerotia, depending on the organism and its life cycle stage. Yet, the source of relative quantitation become irrelevant because gene-expression analysis tools (including DeSeq.) are developed to analyze bulk populations—containing millions of cells—. Consequently, cell number differences between the treatment (Corn+AF70) and control (PDB+AF70) are small-uncontrolled inputs when equivalent sequencing depth has been achieved. Different cell counts between the treatment and the control of gene expression studies is therefore not a factor. We intend to use EDNAtran in metatranscriptomic analyses, where cell counts of organisms are difficult (or impossible i.e. unculturable-unknown organisms). Therefore, EDNAtran relies on a good quality sequencing data and equivalent sequencing depth to be able to differentiate between high and low-frequency hits.

The use of replicates in gene expression analyses using NGS are crucial, yet, this study used one replicate for all four RNA sequencing samples. In a real case scenario—where samples potentially containing *A*. *flavus* are collected—obtaining high-quality mRNA libraries is more important than replicates. This study produced twelve RNA extraction samples from which four RNA extractions having the highest RNA integrity and quality (RIN≥8) where selected to be sequenced. Replication will be needed for statistical hypothesis-driven research, but it is not necessarily required for presence/absence queries or for the development of e-probes. Sequencing depth and sequencing quality equivalence are the most important metric for diagnosis.

Future studies need to include multiple blind samples to assess the usefulness of the new EDNAtran protocol to indicate the active production of aflatoxin. In this study, we have shown that in a known positive transcriptomic database, EDNAtran is capable of discriminating between production and no-production of aflatoxin. However, blind samples will provide a realistic assessment of the tool.

## Supporting information

S1 Table Expression values (log2 fold change) for AF70 *A. flavus* strain for two culture conditions (ground corn and PDB). For each Gene ID, expression levels are listed along with p-values.
(XLSX)
